# Acute Borrelia infection inducing an APMPPE-like picture

**DOI:** 10.1186/s12348-016-0088-x

**Published:** 2016-06-13

**Authors:** Munjid Al Mousa, Frank Koch

**Affiliations:** Vitreoretinal Unit, University Eye Hospital, Frankfurt/Main, 60590 Hessen Germany

**Keywords:** APMPPE—acute posterior multifocal placoid pigment epitheliopathy, Lyme disease—Borreliosis, Borrelia

## Abstract

Acute posterior multifocal placoid pigment epitheliopathy (APMPPE) is an uncommon disorder of unknown etiology affecting the retina, the retinal pigment epithelium, and the choroid. Although several etiological factors have been suggested, none has been confirmed. We report a case of APMPPE associated with acute infection of Borreliosis. A 30-year-old man presented with a decrease in vision in the right eye of about 1-week duration. His visual acuity in the right eye was 6/36. Fundus exam revealed the presence of multiple placoid creamy retinal/subretinal lesions in the right eye. Fundus fluorescein angiography supported the diagnosis of APMPPE. Blood tests revealed the presence of concomitant acute Borreliosis infection, as confirmed by IgM. The patient received oral prednisone therapy and amoxicillin. Six weeks later, the visual acuity returned to 6/6, and the patient was symptom free. Borreliosis can have several manifestations in the eye. One of the less common presentations is an APMPPE-like picture. The clinician should suspect acute Borreliosis infection in patients presenting with APMPPE, especially when there is a history of a tick bite, when the patient has systemic symptoms, or when living in/visiting endemic areas. This may help in the prompt management of APMPPE, avoiding complications due to the condition itself, or systemic involvement secondary to the Borreliosis infection.

## Findings

### Introduction

Borreliosis (Lyme disease) is an infectious systemic disease caused by bacteria (a spirochete) of the Borrelia family. It is transmitted by the bite of a tick of the genus *Ixodes* [1, 2]. It may cause a systemic infection, involving the skin, joints, heart, nervous system, and sometimes the eye [3]. Eye involvement may include conjunctivitis, uveitis, keratitis, optic neuritis, involvement of multiple cranial nerves, and pars planitis [4]. In this case report, we describe a patient that presented with acute posterior multifocal placoid pigment epitheliopathy, and was found to have a concomitant acute Borreliosis infection as confirmed by IgM levels. Although a relation between APMPPE and Borreliosis had been previously suggested in the literature, this association has not been proven. In our case report, we explore this relation further.

#### Case

**Fig. 1:**
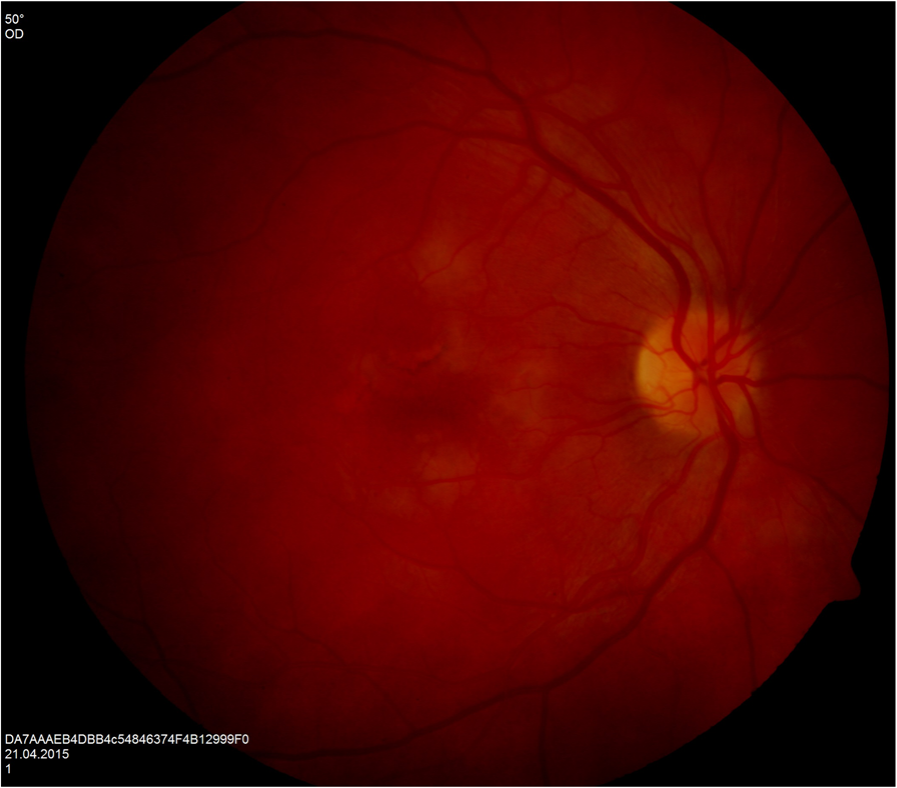
Color fundus photograph showing multiple creamy placoid lesions involving the posterior pole of the right eye, consistent with a diagnosis of APMPPE

**Fig. 2:**
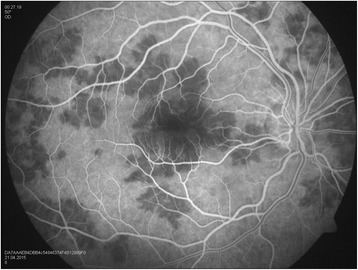
Fundus fluorescein angiography of the right eye showing early hypofluorescence of the lesions

**Fig. 3:**
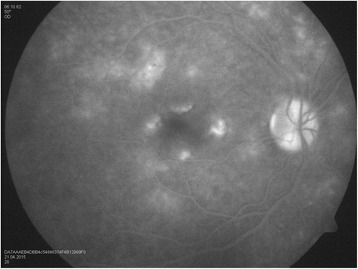
Fundus fluorescein angiography of the right eye showing late hyperfluorescence of the lesions

**Fig. 4:**
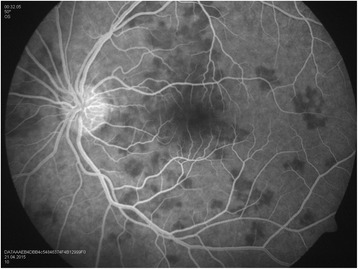
Fundus fluorescein angiography of the left eye showing early hypofluorescence of the lesions

**Fig. 5:**
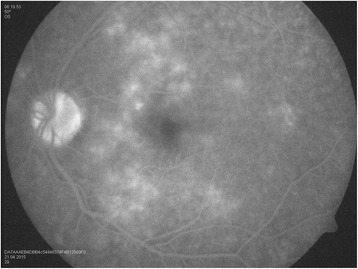
Fundus fluorescein angiography of the left eye showing late hyperfluorescence of the lesions

**Fig. 6:**
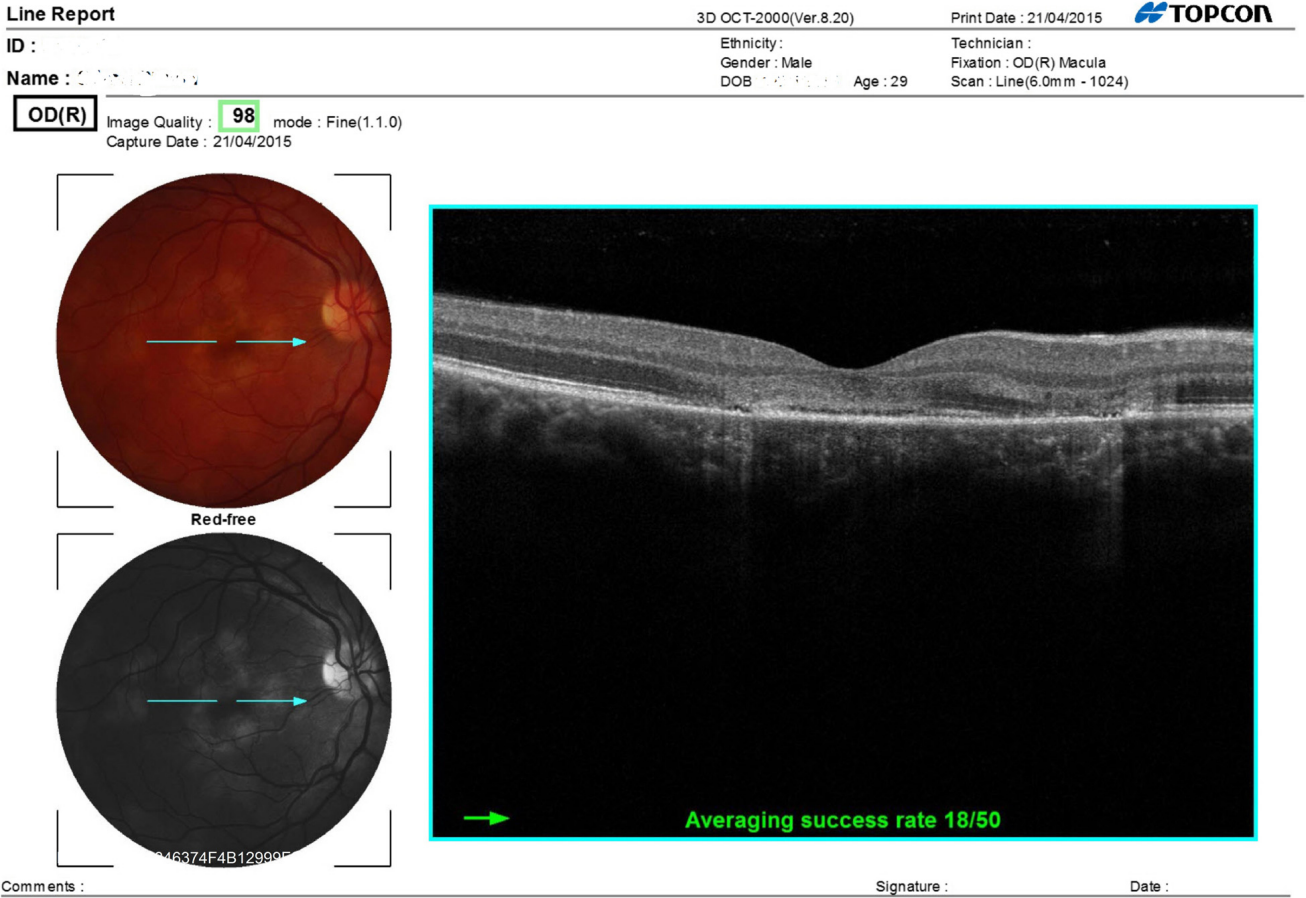
OCT image of the right macula showing disruption of the inner segment/outer segment (IS/OS) junction and minimal subretinal fluid

**Fig. 7:**
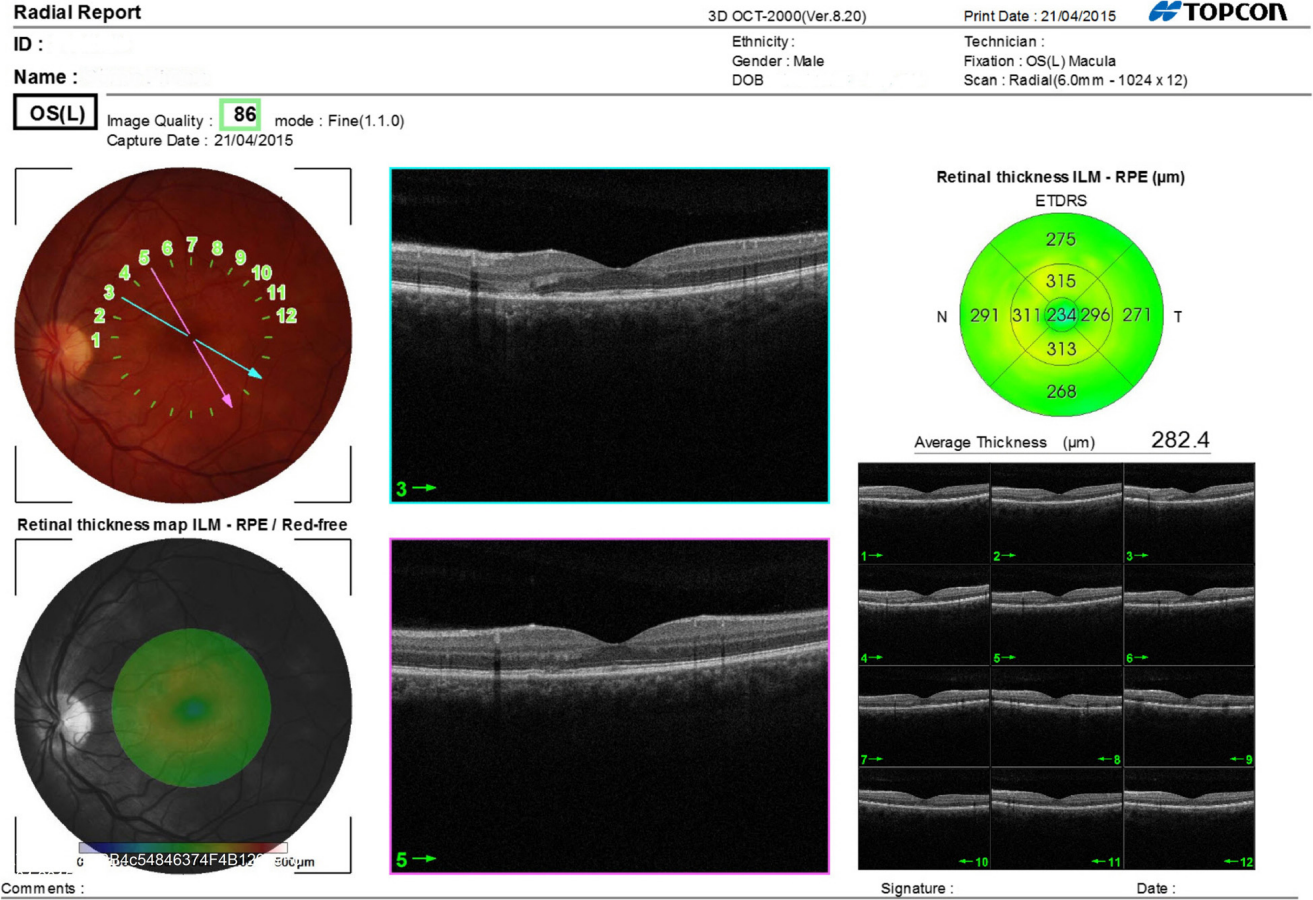
OCT image of the left macula showing minimal involvement in comparison with the right eye

Our patient is a 30-year-old man, with no history of medical problems, which presented with bilateral gradual blurring of vision of about 1-week duration. He had no photopsia, no eye pain, no floaters, no diplopia, and no perceived visual field defects. Furthermore, he had associated mild headache, neck stiffness, and general malaise, but no arthritis or fever. On ophthalmic exam, his visual acuity was 6/36 in the right eye and 6/6 in the left eye. There was no associated relative afferent papillary defect. The anterior segment examination was unremarkable with no inflammatory cells in the anterior chamber and no lens opacities. The intraocular pressure was within the normal range. Upon retinal exam, he was found to have multiple creamy yellow-white placoid subretinal lesions in the right eye, without vitreous cells, mainly at the posterior pole (Fig. 1). The optic nerves were normal. Fundus fluorescein angiography revealed early hypofluorescence (Fig. 2) and late hyperfluorescence (Fig. 3) of the retinal lesions. The left eye also showed similar angiographic findings (Figs. 4 and 5). The clinical findings, supported by the fundus fluorescein angiography findings, led to the diagnosis of APMPPE. Due to the vague systemic complaints and the patient himself being an avid athlete who spent most of his time outdoors, we ordered a few systemic blood tests, one of which was Borreliosis titer. IgM titer (Elisa and Immunoblot) for Borreliosis turned out positive, implying acute infection, despite the fact that the patient did not remember any insect/tick bite or skin lesions. Ocular coherence tomography (OCT) was also done, and it revealed the presence of macular involvement in the right eye (Fig. 6). This involvement was manifested as a disruption of the inner segment/outer segment (IS/OS) junction and minimal subretinal fluid. On the other hand, the OCT of the left eye showed minimal involvement of the fovea (Fig. 7) in comparison with the right eye, which could explain the preservation of vision in the left eye. In light of the macular involvement in the right eye, we suggested the use of oral steroids (80 mg prednisone) to be tapered gradually. We offered the use of intravenous ceftriaxone for the treatment of the Borreliosis infection, but the patient refused since he was a professional athlete and was not keen on receiving intravenous therapy, as it may delay his training. Doxycycline was not considered, due to the risk of phototoxicity. Eventually, the patient agreed on taking oral amoxicillin 500 mg three times daily for 3 weeks.

Six weeks after taking the treatment, the patient’s visual acuity improved to 6/6 in both eyes and the retinal lesions completely resolved. The patient tolerated the treatment well with no side effects.

### Discussion

Acute posterior multifocal placoid pigment epitheliopathy (APMPPE) is a rare inflammatory condition of unknown etiology affecting the retina, the retinal pigment epithelium, and the choroid [5, 6]. It was first described by Gass in 1968. It was hypothesized back then that APMPPE may be caused by an infectious agent. However, this could not be proven. The pathogenesis till now is still not absolutely clear, but it is speculated to involve inflammation and ischemic changes. For the diagnoses to be made, a high index of suspicion is needed, based on the clinical picture mainly and supported by fundus fluorescein angiography findings. Although a relation between APMPPE and Borreliosis had been suggested in the literature, this association was never confirmed. And since APMPPE is an uncommon condition, no major study was done to explore its association with Borreliosis. The biggest study that we were able to find was by Wolf et al. in 1992 and it included 18 patients. None of the patients with APMPPE in this study had positive antibodies against *Borrelia burgdorferi* [7]. As a result, the investigators concluded that they were not able to establish an association between these two conditions. However, a few case reports were published since then that showed that this association may actually be true. We found a case report by Augsten et al. in 2009 [8] and another in early 2015 by Wang et al. [9] that suspected this association. Our case report, to our knowledge, will be the second from the year 2015 to be published in the literature, and the fifth to be reported from Germany [10–12]. Furthermore, it seems curious to us that most of the above mentioned reports that suggest a link between the two diseases come from Europe, and that there are multiple bacterial species causing Lyme disease in Europe (*Borrelia afzelli*, *Borrelia garinii*, and *B. burgdorferi*), while in America the disease is mainly caused by one species only (*B. burgdorferi*) [13, 14]. Whether this observation is a coincidence or not is yet to be determined.

Our case report is not the first to explore the relation between Borreliosis and acute posterior multifocal placoid pigment epitheliopathy. Although these reports are few in number, they do suggest a possible association that is worth further investigation. If such an association could be proven, even in a minority of patients, this would confirm the importance of setting new recommendations and guidelines that may help the clinicians in the management and treatment of patients who suffer from APMPPE and/or Borreliosis. We would also like to emphasize on the importance of suspecting acute Borreliosis infection in patients presenting with APMPPE, especially if there is history of tick bite, presence of systemic symptoms, or living in/visiting endemic areas.
